# Group medical visits can deliver on patient-centred care objectives: results from a qualitative study

**DOI:** 10.1186/1472-6963-13-155

**Published:** 2013-04-29

**Authors:** Josée G Lavoie, Sabrina T Wong, Meck Chongo, Annette J Browne, Martha LP MacLeod, Cathy Ulrich

**Affiliations:** 1 University of Northern British Columbia, School of Health Sciences, TLC building 10-3516, 3333 University Way, Prince George, BC V2N 4Z9, Canada; 2 University of British Columbia Centre for Health Services and Policy Research, Vancouver, Canada; 3 University of British Columbia School of Nursing, Critical Research in Health and Health Care Inequities, Vancouver, Canada; 4 University of Northern British Columbia, School of Nursing, Prince George, Canada; 5 Northern Health Authority, British Columbia, Prince George, Canada

**Keywords:** Primary health care, Clinical encounter, Self-management, Effectiveness, Chronic diseases

## Abstract

**Background:**

Patient-centred care emerged in the late 1960s as a framework to guide providers and decision-makers towards the provision of more effective health care and better outcomes. An important body of literature has since emerged, reporting mixed results in terms of outcomes. To date, assessments of the effectiveness of patient-centred approaches have focused one-on-one consultations. The purpose of this article is to explore dimensions identified as key in the patient-centred literature in the context of primary health care services delivered in a group setting. Group Medical Visits (GMVs) offer a novel format for the delivery of patient-centred primary health care services, especially for patients living with complex morbidities.

**Methods:**

Drawing on a large study of GMVs, we report on key format and process-oriented elements identified in GMVs, and on their link to improved outcomes. For the purpose of this study, we interviewed 34 providers and 29 patients who have been engaged in GMVs, delivered in rural, northern and First Nation communities in British Columbia, Canada.

**Results:**

Our analysis shows that the delivery of PHC in a group format results in a shift in the role of the provider, from that of an adjudicator involved in imparting norms of self-care, to that of a facilitator who assists the group in defining norms of self-care that are based on medical knowledge but also on the broader context of patients’ lived experience and on their pragmatic experience. In a group process, peer-patients take on the role of promoting these norms to other patients. This results in a significant shift in the role of the provider, increased trust, increased knowledge for the providers and the patients and better patient self-management. Our results also show increase satisfaction for patients and providers.

**Conclusions:**

GMVs offer an alternative format for the provision of PHC that brings together the benefit of a group process and of a clinical encounter. This format can successfully deliver on the promises of patient-centred care.

## Background

Past conceptualizations have framed the clinical encounter (CEs) as a one-on-one interaction between a patient and a provider, focused on the provision of care for acute and chronic episodes of illness [1], pp. 1087–88, [2], p. 3, [3]. The provider was understood as having the primary responsibility of evaluating, diagnosing, treating the patient’s condition, while exercising independent judgment and acting in the patient’s ‘best interest’. Conceptualizations of the role of the patient, most notably Parsons’ “sick role”, described the patient as being released from social norms yet obligated to seek care. This role was however largely passive in regard to making decisions, and subservient to the “expert” knowledge of the provider [1].

This conceptualization of the CE has been broadly criticized for oversimplifying the CE, for assuming that patient values and preferences are in tune with the practitioner’s [4], and for framing the role of the patient as that of a passive recipient of information [5]. Boyer and Lutfey have eloquently argued that while this conceptualization might have aligned with reality in the past, the patient-provider relationship has changed considerably over the past 50 years, as a result of recognizing the significance of social contexts in shaping patients’ individual experiences; the expansion in the construct of the “patient” from one experiencing an acute episode to include those managing chronic conditions and those at risk of developing a condition as a result of surveillance technologies; and patients’ ability to independently access information to actively engage in and/or challenge decision-making related to their treatment [6].

Along with this conceptual shift, there has been increased scrutiny on patient-provider communications in the context of the CE. Research in medical anthropology, sociology, psychology, economics and health services research have focused on patient-provider interactional processes, and on the link between these processes and outcomes. An extensive body of literature has emerged over the past three decades, aiming to inform an “ideal CE” that might result in better outcomes for patients. The “patient-centred” approach emerged in the late 1960s. Mead and Bower’s review of the literature [7,8] identified the following key criteria for a patient-centred CE: 1) exploring both the disease and the illness experience (biopsychosocial perspective); 2) understanding the whole person (patient-as-person); 3) finding common ground regarding management (sharing power and responsibility); 4) incorporating prevention and health promotion (the therapeutic alliance); and 5) enhancing the doctor-patient relationship (the doctor-as-person) [8], pp. 1087–88]. Within this framework, providers are called upon to understand the social and family context, culture and history of their patients. Providers and patients are expected to interact in ways that are non-biased, demonstrating understanding and acceptance of the other’s potentially diverse background [9].

Recognizing that individuals are situated within a broader social matrix [10], the literature on patient-centred care acknowledges how power differentials can affect the CE [11,12] and emphasizes how leveling power relations can help patients feel safer, share their healthcare needs, accept the information provided and incorporate this information into their own daily lives [13,14]. Pilnick and Dingwall have argued that power asymmetries are inherent to the physician role: “Physicians… are not just healers of the sick…, but also socially licensed adjudicators on contested or contestable claims to dependency. They are required to rule on both the ‘facts’ of the case and on the motivation of the patient” [15], p. 1380]. This dual role, we argue, has the potential to undermine the objectives of the patient-centred project in one-on-one CE. A 2001 Cochrane review on the link between the patient-centred approach and health outcomes has been mixed [16]. Interestingly, to date, assessments of the effectiveness of patient-centred approaches have been in the context of one-on-one consultations. Format elements such as the length of the consultation, the presence of family members or other potential advocates, or providers’ payment scheme (salaried or fee-for-service) have not been documented in the research on patient-centred CEs.

In our research, we have identified that Group Medical Visits (GMVs) can provide comprehensive PHC services in a manner that addresses some of the shortcomings of the one-on-one CE. While delivering health services using a group format is not new, the delivery of PHC services using GMVs is relatively recent. In British Columbia (BC), Canada, GMVs are emerging as an innovative format for the provision of primary healthcare [17-19] in areas underserved by PHC providers and for populations living with co-morbidities. The literature identifies two types of GMVs: (1) “homogenous” GMVs where those in attendance share a diagnosis (diabetes) or concern (living with chronic pain) (Cooperative Healthcare Clinic, CHCCs; and Physicals and Shared Medical Appointments, PSMAs); and (2) “heterogeneous or mixed” GMVs which are a drop-in format where some teaching may be shared (Drop-In Group Medical Appointments, DIGMAs) [17,18]. In GMVs, primary healthcare (PHC) services are typically delivered to a group of 12–20 patients by their regular provider (e.g., family practice physician, nurse practitioner). They range in length anywhere from 60–120 minutes with the average being reported as 90 minutes. Other healthcare providers, and sometimes representatives from community organizations (the Canadian Diabetic Association, for example), may be involved. The content of GMVs often includes the provision of medical care related to a common chronic condition (e.g., medication adjustments for diabetes management), screening and early detection of health conditions, or routine physical examinations, and a health education or health promotion component [20].

The purpose of this article is to identify and describe key format and process elements used in GMVs as identified by providers and patients engaged in GMVs, and explain how these key elements link to improved health outcomes. To do so, we first turn to the work of Kurtz [21] on group-delivered care. We then discuss format and process elements, and finally link those to outcomes reported by both providers and patients.

### Framework for understanding GMVs

To better understand the potential role of GMVs in shaping CEs, we draw on the work of Kurtz [21] who explored the role of professionals in self help and support groups. *Self-help groups* such as Twelve-Step groups, gather to explore and exchange experiences and information on a common problem. They are generally change-oriented, and the change sought may be personal or societal or both. Professionals may be invited but peers provide the main guidance. *Mutual support groups*, such as those for parents of autistic children, meet to share and find information. Change is not a main focus although positive management of the common problem is an obvious objective. In both self-help and support groups, the group plays an important role in terms of information sharing and mutual support. However, group interactions are not intended to be therapeutic. In contrast, *psychotherapy groups* are professionally-led, with group interactions designed for a therapeutic purpose, one of which could be to create opportunities for the development of basic social skills. In the context of these three types of groups, the role of professionals is circumscribed to that of a source of information (self-help and mutual support group) or as an authority leading the group and ascribing meaning to interactions between group members (psychotherapy).

GMVs are a hybrid format. They are comprised of PHC services that are professionally-facilitated, and incorporate aspects of both a CE and a social group. Past work has suggested that incorporating the support group concept into the CE is important [6,22]. These authors suggest that group support is a socially contextualized experience where power relations are somewhat more leveled because of the absence of clinical providers. Research suggests that support groups function with specific structural elements including voluntary participation, and an informal context with a flexible, non-hierarchical, non-expert, non-medical, and power-sharing model [23-25]. Included as well are process-oriented elements such as: empowerment, advocacy for group members, reciprocity, the affiliation with peers with similar life experiences, catharsis, the general understanding that one can experience self-healing as a result of being helpful to other group members, and the sense of community [26,27]. We argue that in the context of GMVs, the CE is co-produced by providers and patients. The benefits of the “group function” can be fostered (as opposed to undermined) by the active participation of a medical provider’s role – particularly because the conventional role of medical provider as adjudicator (with attendant power differentials) are mitigated by the group function and process. Drawing from this work, we identify format and process-oriented elements that are related to group processes and can inform our analysis of GMVs. We argue that providers and patients’ perspectives are key to understanding the contribution of the GMV-based CE. We also note that the express purpose of the GMV is to produce change in patients’ self-management. Accordingly, any discussion of GMVs should attempt to address GMVs’ ability to produce this sought outcome.

## Methods

The analysis presented in this article draws on a larger mixed-methods study of GMVs conducted in several rural communities in the Canadian province of British Columbia (BC). The purpose of the larger study was to examine the impact of GMVs on the quality of PHC on-reserve and in northern communities, from patients’ and providers’ perspectives. Interview data were collected on patients’ and providers’ perspectives on the access to and quality of PHC provided through GMVs. Approval was received from the University of British Columbia Research Ethics board, the University of Northern British Columbia Research Ethics Board and the North Health Research Review Committee.

Sampling was purposive [28] and theoretical [29] in order to capture a wide variation of views. Eligible providers were those who had taken part in delivering GMVs during the previous year. Providers identified possible patients. Eligible patients were those aged 19 years and over and who had attended one or more GMVs over the previous year. Patients who consented to being contacted were mailed a letter. One week later, potential patient participants were contacted by a team member by telephone. After signed informed consent was obtained, participants were interviewed by a research team member. Open-ended questions explored participants’ experiences in facilitating or attending GMVs. Questions addressed critical components in the provision of a successful GMV, information on any barriers to delivering or receiving PHC in a group format, and recommendations to improve the delivery of GMVs for other providers or patients. At the end of each interview, participants were given $15 in appreciation of their time.

Interviews were held in nine different rural communities, ranging in size from 700 to 80,000 people. The communities were chosen based on guidance from our Health Authority partner. Members of the research team directly observed providers delivering a GMV. All interviews were digitally recorded. The recordings were transcribed and cleaned of any personal identifiers. Transcripts were first compared with the audio recordings for technical accuracy. Using interpretive thematic analysis for qualitatively derived data [30], the team reviewed the transcripts to identify linkages to theoretical perspectives as well as any recurring and contradictory patterns. A data coding scheme was developed, validated through independent coding and organized using Atlas TI software. Coding categories were refined by at least two team members and an audit trail of analytic decisions maintained as data collection continued. Having also considered alternative interpretations, a conceptual representation with full descriptions of the themes reflected in the data was developed. Research team members and PHC experts evaluated and discussed credibility of the analysis and feedback on the aggregated results was obtained from participants [31].

A total of 63 participants completed an in-depth interview to provide their experiences with GMVs. Providers who were interviewed included family physicians (n=10), nurses (n=7), nurse practitioners (n=2), PHC coordinators (n=4), other allied health professionals (n=11), such as nutritionist and social workers and supportive personnel, such as medical office assistants and community health representatives, who were involved in delivering a variety of GMVs (see Table 1). Table 2 shows that patient participants (n=29) were an average age of 62 years, mostly female, and married. Patients reported being either Caucasian (55%) or of Aboriginal descent^a^, where most were First Nations^b^ (41%). Almost half of patient participants reported a household income of less than $30,000 CDN.

**Table 1:** Demographic characteristics of providers (n=34)

**Type of provider attending GMV (n)**	
• Family physician	10
• Nurse	7
• Nurse practitioner	2
• Primary healthcare coordinator	4
• Other (includes medical office assistant, community health representative, outreach coordinator)	11
Number of GMVs delivered in one month	
• Mean (SD)	1.4 (1.9)
• Range	1-6
Type of GMVs delivered (%)+	
• Cooperative Health Clinic model/Homogenous	88.6
• Drop-in Group Medical appointments/ Mixed	34.3

Note. +providers were asked to list all types of GMVs delivered.

**Table 2:** Characteristics of patients (n=29) attending group medical visits

**Age (years)**	
• Mean (SD)	62.0 (16.0)
• Gender (% female)	65.5
Self-reported health (1–5)+	
• Mean (SD)	2.8 (1.1)
Ethnicity (%)	
• Caucasian	55.2
• Other	0
Aboriginal (%)	
• First Nation	41.4
• Métis	3.5
Marital Status (%)	
• Married	79.3
Income (%)	
• <$20,000	37.9
• $20,000-$29,999	20.7
• $30,000-$39,999	20.7
• $40,000-$49,000	3.5
• $50,000-$59,999	6.9
• $60,000-$69,999	3.5
• ≥$70,000	0.0
• Missing	6.9
Number of chronic conditions (%)	
Range	0 – 7
• 0	10.3
• 1	6.9
• 2	27.6
• 3 or more	55.2
Chronic conditions (%)++	
• Diabetes	58.6
• Arthritis	48.3
• High blood pressure	51.7
• Depression	34.5
• Heart Disease	20.7
• Other: Kidney Disease	10.3
• Other: Cholesterol	6.9
• Other*	27.6
GMVs attended in the last year	
• Range	1-15
• Mean (SD)	4 (3.0)
Type of GMV attended (%)	
• Cooperative Health Clinic model/Homogenous	82.8
• Drop-in Group Medical Appointments/ Mixed	17.2
Satisfied with care from family physician (%)	
• Always/Usually	79.3
• Sometimes/Rarely/Never	20.7

Notes: +higher score=better health; ++patients were asked to report all chronic diseases where they were given a diagnosis.

Patient participants attended an average of four GMVs in the previous year; 24 patients attended a homogenous GVM where all in attendance shared a similar diagnosis (pain or diabetes, for examples) and 5 attended a heterogeneous GMV where diagnoses were mixed. Just over half (n=16) reported having three or more chronic conditions, with the three most common conditions being diabetes (59%), high blood pressure (52%), and arthritis (48%).

## Results and discussion

Overall, the patients and providers we interviewed reported a great deal of satisfaction with the GMV format. The following section summarizes key results. We divided these results into three broad categories: key format elements, process-oriented elements and outcomes. We draw on the discussion of Kurtz to inform this analysis. Excerpts are interwoven to illustrate these key elements and outcomes.

### Key format elements

Providers and patients shared a number of elements that they portrayed as key to the success of GMVs. We organized these into 3 sub-categories: a) Social event; b) Affiliation; and c) Co-production of the GMV. Each will be discussed in turn.

a) Social event: Patients and providers emphasized the importance of the social component of the GMV. In the quote below, a provider linked the relaxed atmosphere of the GMV to being able to gather important medical information, and information about the broader context of patients’ everyday life:

“I think that’s a big thing having a group where they can come in and be seen, when they need to be seen. Interaction, I think the social aspect of it is important for people, it’s like meeting old friends and there are some people who might be with their chronic illnesses they can’t get out very much, they spend a lot of time at home. I think that’s a good aspect to them, they love coming in, having a cup of coffee with their friends and just talking about things. And, of course, then having their conditions checked. I think there’s this level of comfort too for them, they come in, they know they’re being seen, they’re feeling that they’re being really well looked after. Because for some of these people, especially the chronic patients, [the GMV] gives them a bit of peace of mind” Provider #6.

b) Affiliation: Both providers and patients highlighted that the social element results in a shift in power, in part because of the presence of peers with shared experiences, but also because providers share the role of adjudicator with patients attending the GMV. All stated that this was a positive outcome. Many participants reported that GMVs provided flexibility and a “leveled playing field” in which to interact with each other. None of the providers we interviewed portrayed this shift of power negatively. The quote below, reflected the general sentiment expressed.

“With a group you have a feeling of being part of many, whereas when I’m here with you or with my doctor, or one-on-one, quite often you’re intimidated by someone who knows more than you do and it’s just a feeling sometimes of isolation and loneliness because you have the disease and it’s a different feeling completely. And I feel a lot more comfortable in groups than one-on-one” Patient #16.

Both providers and patients suggested that the group acts as a community in which participants share common experiences. Telling one’s story was reported as valuable because of its cathartic potential, and also because it creates a way to connect to others with similar experiences. This was highlighted as beneficial.

“It seems like people do get to share and feel heard in that setting very well and very thoroughly. There’s this real sharing of stories that I’ve only occasionally heard about… where the actual telling of your story and the recounting and how you tell yourself your story and reinterpreting it is actually a big part of therapy” Provider #20.

“And that sharing in itself is therapeutic to some degree really, you know, if you have a listener or all these people” Patient #16.

d) Co-production of the GMV: Providers highlighted key differences between the one-on-one and the GMV format, in that the GMV is co-produced by the provider(s) and the group. Co-production was achieved by providers giving space for the group process to become a central component for health education. Since a positive group process was seen as key to the success of the GMV, providers reported that they actively sought feedback on the GMV format from patients.

“I think care providers have the real instinct to lecture and it’s [the GMV] totally not about that… Talking at the group is way more about not being the expert and letting the group be the expert and then correcting people if misinformation gets out there” Provider #13.

“We had to shift things around. So what has happened from that point on is it’s patient driven. How did you like today? Would you like it a little different next time? You [patients] help us plan. So the patients always were the partner in developing the group, this is what we would like next. But they always knew that they could get their [individual] doctor’s visit that day that was one thing that stayed stationary” Provider #29.

Providers also reported that GMVs provided them unique learning opportunities.

“I think that it [the GMV] has helped me to be more creative in looking at ways to meet people’s needs. Some of that just comes from the patients themselves because they often have some really neat ideas about how to overcome challenges or difficulties in dealing with the diabetes. So I think that, not only have I become more aware but I’ve also, they’ve given me some really good tips and ideas. I think there’s stuff I learned that I wouldn’t have learned if I had done it on an individual basis. There’s a lot of value that comes out of that, that kind of impromptu patient teaching of each other” Provider #28.

### Key process-oriented elements

Providers and patients shared a number of process-oriented elements linked to the format elements reported above. We organized these into 4 sub-categories: a) Safety; b) Mutual support; c) The normative effect; and d) The changing role of the provider. Each is discussed in turn.

a) Safety: a key theme that recurred in patients’ interviews was that they felt safer when in a group than in a one-to-one clinical encounter. One patient commented:

“If you have a group medical visit on a particular subject there’s a certain protection there in numbers too, I mean there’s probably not going to be a whole lot of ‘in your face’ and things done to you or maybe even more probing questions but, you always remain somewhat of anonymous in a group. A little bit more than just one-on-one, if it’s going to be in a group medical visit you might be safer, you might not be probed, poked quite so much” Patient #16.

This was recognized and acknowledged as positive by providers as well.

b) Mutual support: One reason for feeling safer was the presence of others to advocate when miscommunication occurred:

“You know we try to support one another, it’s kind of human to do that. It’s human to have compassion for other people who have problems and you can show that and you can feel that from other people when you’re in a group, you don’t in isolation. Oh yeah, [in a GMV] it feels great, it feels really good, it feels like I have advocates, I have support, I have people, a group of people that will just come to your rescue if you needed to be rescued they’re just there to really help you along, you know. It’s a great feeling, groups are really advantageous” Patient #17.

c) The normative effect: While many patients reported feeling safer in a group, many also reported instances where members of the group intervene in a patient’s care strategy, pressuring them to take better care of themselves. The group thus served a protective role against providers being “*in your face*”, but was also more effective in promoting norms of self-care.

“And then all of a sudden someone else will start telling well it’s because you’re not doing your diet stuff, you know, start giving them heck, right, so, um, so it was a very, very, very powerful for people to see their own numbers within the context of the group and it was almost I think most humans are kind of competitive and they look at it and say man I want, I want to do better than that” Provider #1.

“So many people use denial. And so when you’re in a group, I think it just changes that dynamic and you have to face up to the other people, you know what? I never do my blood sugars, I don’t take care of myself so that’s a different thing than you’re private about with the doctor and then admitting something in a group” Provider #23.

Whereas patients related negative experiences when, in a one-on-one consultation, providers attempted to impress upon them the need to change behaviours (i.e. “*He’ll give it to you in the office*” [Patient #20]), none of the patients we interviewed portrayed peer interventions negatively.

d) The changing role of the provider: Thus, the group was reported as playing a normative role in the creation of shared norms of self-care behavior. This was associated with a shift in the role of the provider, from that of an expert tasked with defining norms of behavior (the adjudicator role described earlier) and imparting these norms to the patient (as in psychotherapy groups), to that of a facilitator of a group process. Two patients commented on how this shift in the role of their providers resulted in increased trust:

“I’ve learned to trust him. I trust him more than I used to and that’s important, that bond of trust has to be there. I trust him more when I see that he’s open to learning and figuring out new things that are only happening in group dynamics” Patient #8.

“Do you know…what [GMV] helps me to see is what the physician, his devotion of trying to solve a health problem and trying to correct it. That actually re-establishes my faith in the medical system because you can see that they’re really devoted to trying to figure out really what is ailing you” Patient #13.

In contrast, providers noted that the social context of the GMV allowed patients to talk about their condition within the context of daily life. As one provider explained, providers can develop a better understanding of patients’ lived experience, and of barriers to adherence:

*“I think it was just understanding, when you sort of follow the kind of dialogue that goes on in the group just realizing how much other factors were involved in people’s lives, that made diabetes not a priority right? And so whether that was, pain or things going on in their life or, whether its sadness or disappointment or bitterness about the way diabetes sort of affected their lives and their choices, food choices. And that people were still struggling with integrating it into their life, right? I think just understanding those things a little bit better and just to be able to express those things seemed to be helpful,…because often otherwise it would be sort of fruitless dialogue about your numbers aren’t where they are, and then there would be, you have to take other medication. Yes, I’m doing this, and people just wouldn’t step up to the things that they were doing or not doing. It seemed to be easier to get to the roots of****why****in the group visits, because obviously for people either it wasn’t a priority or they were still just really upset about it. Or they had lots of excuses about why they couldn’t do the daily exercise” Provider #15.*

This quote also shows that the provider gained insights into the factors that affect patients’ day-to-day lives and how this, in turn, affected patients’ ability to self-care on an everyday basis. Another provider offered a slightly different view, emphasizing how power relations remain between the provider and patients in a GMV, but how this is somewhat hidden as a result of group dynamics:

*“It [the GMV] creates an environment that is the****trickery in medicine****- to think people are having a social gathering and you’re working the crowd and doing the medical work while they’re having a good time, I mean that’s optimal, right, that’s optimal medicine so there’s no fear involved, there’s no worry, people are enjoying themselves, it’s almost social like and yet there’s a team going around getting all the information that needs to be gleaned from this group of people and that’s the secret, so you turn it into a really positive experience for the patients so that’s why they want to keep coming back because I mean if you’re going to go see your doctor and you could sit and have a cup of coffee, it’s an enjoyable conversation, people talk about a lot of topics” Provider #1.*

The idea that the social element acts as a form of *trickery*, while noteworthy, was not echoed by other providers or by patients.

### Outcome

Key format and process-oriented elements create a different environment for the provision of PHC. While this study did not document clinical outcomes, we did document proximal outcomes that have been identified in the literature as key to the provision of effective PHC: a) Better information sharing; and b) Better self-management.

a) Better information sharing: At an individual level, both providers and patients reported therapeutic benefits to the process of storytelling. One provider commented:

“I think when you have a group around the table listening somehow…it doesn’t take as long to get out what you need to get out because there’s more soaking up of the story going on or something. It seems like people do get to share and feel heard in that setting very well and very thoroughly. There’s this real sharing of stories that I’ve only occasionally heard about… where the actual telling of your story and the recounting and how you tell yourself your story and reinterpreting it is actually a big part of therapy” Provider #20.

A patient commented:

“And that sharing in itself is therapeutic to some degree really, you know, if you have a listener or all these people” Patient #16.

Beyond the cathartic value of sharing one’s story, providers commented that patients’ storytelling provides invaluable teaching material for others:

“For some of the patients who came who are struggling with trauma issues and low educational levels and all, it seemed to be a great fit because, as opposed to other ways of getting information like written information or, information in a really power imbalanced setting with a provider and you in an individual visit, I think it was a much more comfortable, accessible open setting for people to try and understand what they needed to understand. I think it’s an ideal sort of format for marginalized people” Provider #15.

Some patients also highlighted the synergistic value of storytelling in a group format, and how the story becomes a stepping stone for teaching and learning:

“I think for most people they do their best when they’re within a group or within a setting where there’s more than one person and the focus isn’t always on the one person. I just think that one person might generate an idea and others can take the spin off that idea. You can get into greater depth with aspects like that. Sometimes if you’re a little too timid to ask the questions maybe someone else will ask them for you” Patient #16.

b) Better self-management: Providers discussed the ubiquity of reciprocal learning within the GMV environment. GMVs allowed for natural opportunistic input into patients’ health issues by providers and other patients. This first quote shows the providers’ perspectives that patients may accept information more readily from other patients than from a provider:

“I think when you’re within a group, whether it means to or not, they do a lot of their own work. They will self-manage with the group. It isn’t just me sitting telling you what to do. They hear from their peers which its, people will change doing something, I could tell them ten times and as soon as somebody beside them with the same condition tells them to do it they listen, they do” Provider #4.

GMV’s key format and process-oriented elements result in an environment that is motivating, helping patients gain the necessary skills, health behaviors, and confidence to manage their own condition:

“I think you come out of the group feeling much more self-confident and willing to challenge your self-management program, you just feel that your batteries are recharged and you can really go after it, till the next group…you want to do more yourself and rely less on others. And say ‘well I think I can do it myself as much as possible’ but then you always realize that there are others out there to help you if you feel you need that help” Patient #16.

“Coming to these group sessions made me realize that I wasn’t managing my diabetes properly and with this information that came from the doctors and other patients together it seemed to make me more aware and to practice better control through the information that I received” Patient #2.

“Well when you can follow the other people and you can see what the doctor is doing for their problems and you can see where you’re because it’s a chronic disease we can see where we’re heading and try to stop it before we get there. We know we’re going to get there eventually but we want to slow down getting there” Patient #19.

We began this paper by arguing that Group Medical Visits are hybrid formats that blend a CE with elements of the support group. Our data has shown that the social context of the GMV does not hinder the effectiveness of the CE, but rather adds value to the encounter by providing opportunities for peer learning and mutual support. Further, the social context of GMVs allows for communication that elicits the patient’s perspective within a social context, and involves patients in choices in order to reach a common understanding of the problem and potential solutions. Our results also show that, contrary to what the self-help literature suggests, the presence of professionals who are actively engaged in the pursuit of clinical objectives, does not undermine the benefit of the group process. Through interaction with patients, providers reported having gained a more advanced communication repertoire, and developed greater self- and situational awareness. The GMV positions the CE into a broader social context, and as a result, providers may be better equipped to deliver more appropriate care for diverse and disadvantaged groups.

Patients and providers reported that the co-production inherent to the GMV resulted in patients buy-in and in providers being more comfortable. Providers reported a better understanding of the patients’ experience and of barriers impeding adherence. They also reported learning from the group. Patients reported feeling safer in a group. They also shared that seeing providers work in a group setting increased their trust in the providers. This resulted in better information sharing, from patients to providers and from providers to patients, and in better self-management.

Patients emphasized the benefit of advocating for each other, especially for some GMV attendees who may be unable or unlikely to advocate for themselves. This type of advocacy may be necessary in order to support those who have suffered historical trauma as they may be apprehensive and unable to seek out the healthcare they require [32]. This can help bridge gaps in cultural understanding (e.g., beliefs about managing chronic conditions) but also affect the nature and quality of service obtained by patients; asking more questions or having questions asked for them facilitates patients becoming more informed about their condition with their particular context. Patients who advocate on behalf of others may also help peers take advantage of a particular treatment approach and draw attention to each other’s contexts for providers [32]. Such advocacy, however, can only be fully realized where trusting relationships between patients, their peers and providers exist.

A key finding is that providers’ normative behaviours, for example, teachings on the need to change one’s diet, are problematized by patients and at times perceived as disrespectful when in a one-to-one visit. The same behaviours are not portrayed as a problem by patients when coming from peers. Patients reported that peer teaching and peer pressure to adopt better self-care strategies were welcomed, and understood as supportive. When such pressures came from providers in a one-on-one CE, the same behavior was portrayed as abusive or threatening.

Our results suggest that GMVs align well with the patient-centred ideal. While power relations between providers and patients remain in GVMs, the group process appears better able to mitigate the impact of power differentials. This is an important finding, and has been perceived as the key limitation to the successful implementation of patient-centred care [7,15,16,33].

Admittedly, the GMV format is not for all patients, nor is it meant to replace all one-on-one visits. This study is limited since data were collected only from rural settings and no data were collected from those who tried a GMV and did not return or from those who refused to attend a GMV. Further work is needed in order to understand which services are best delivered in a GMV format. Still, it is our critical reflection, based on patients and providers’ perspectives, that the GMV aligns with criteria identified as key for patient-centred care. This finding may be especially important for the growing number of patients living with chronic conditions that require ongoing management. The more in-depth and prolonged communication in GMV-delivered PHC create an environment where trust is more likely. As well, this environment provide opportunities for the provider to be sensitized to the social circumstances under which patients became ill and attempt to self-manage [33,34]. The patient's sense of being cared for, as the patients see how committed providers are to achieving patient treatment outcomes, increases trust [35]. Through the GMV, providers can facilitate patient engagement by encouraging them to tell their own story, actively listen, give emotional support, and provide reassurance and information on their condition [36]. This expression of concern and commitment to being of help and the clinical attention to, as well as respect for, social and cultural contexts, can also increase trust in and commitment to the institutions of the larger society [37].

### Conclusions

Our study has shown that the GMV offers visits that reflect the ideal of patient-centred. The GMV answers to the unique exigencies of the CE: “the dynamics of the relationship between healer and sufferer; the heightened vulnerability of the suffering individual; the necessity for clinical responsibility; the need to translate knowledge into individualized or personalized intervention; and the ways in which patient and clinician are connected to larger social and cultural domains of community” [37], p. 411]. The GMV does this by sharing the normative aspect of healthcare between providers and peer-patients. In doing so, the GMV resolves an important paradox faced by providers: that of their dual role of healer and of adjudicator. This dual role contributes to undermining trust.

Finally, the GMV brings out a meaningful understanding of power in the recognition that we all have access to power, and that power can be a resource for positive change. Since patients may not have the skills or confidence to express what they need or want in relation to their care, facilitating empowerment in patients through a GMV environment that allows them to transition from being passive to active participants in their care can be a solution to better patient outcomes in PHC. Through forming relationships of care with their providers, relationships that are voluntary, that respect and enable autonomy, accountability, fidelity and humanity [5], pp. 449], patients are thus able to negotiate care, treatment and support appropriate to their situation and to their attaining the best possible outcomes. While providing a locus for continuity of patient relationships and knowledge, the presence of combined support and care over an extended duration within GMVs can improve patients’ service use and health behavior [38].

## Endnotes

^a^“Aboriginal” is defined in the Constitution of Canada, Section 35 of the Constitution Act 1982, and refers to all Peoples of Indian, Inuit and Métis heritage, regardless of where they live in Canada or whether they are “registered” under the Indian Act of Canada.

^b^“First Nation” is a term that came into common usage in the 1980s to replace the word "Indian". Although widely used, no legal definition of the term exists. The term First Nations people in this paper applies to both Status and Non-Status Indians.

## Competing interests

The authors had no competing interests in the conduct of this research or the preparation of this manuscript.

## Authors' contribution

JL, SW, AB, MM and CU were involved in the design of the study and early conceptualization of this manuscript. JL and SW led the coordination of the study and conducted a large proportion of the interviews. JL, SW and MC analyzed the qualitative data, and drafted the manuscript. All authors read and approved the final manuscript.

## Pre-publication history

The pre-publication history for this paper can be accessed here:

http://www.biomedcentral.com/1472-6963/13/155/prepub
